# Supplementary material for: Simultaneous functioning of different light-harvesting complexes—a strategy of adaptation of purple bacterium Rhodopseudomonas palustris to low illumination conditions
Source: PeerJ. 2023 Jan 31;11:e14769. doi: 10.7717/peerj.14769 (PMC9897067; doi:10.7717/peerj.14769)
Supplement: Supplemental Information 5 [file peerj-11-14769-s005.pdf]

==== Shimadzu LCsolution Analysis Report ====

<3D Graph>

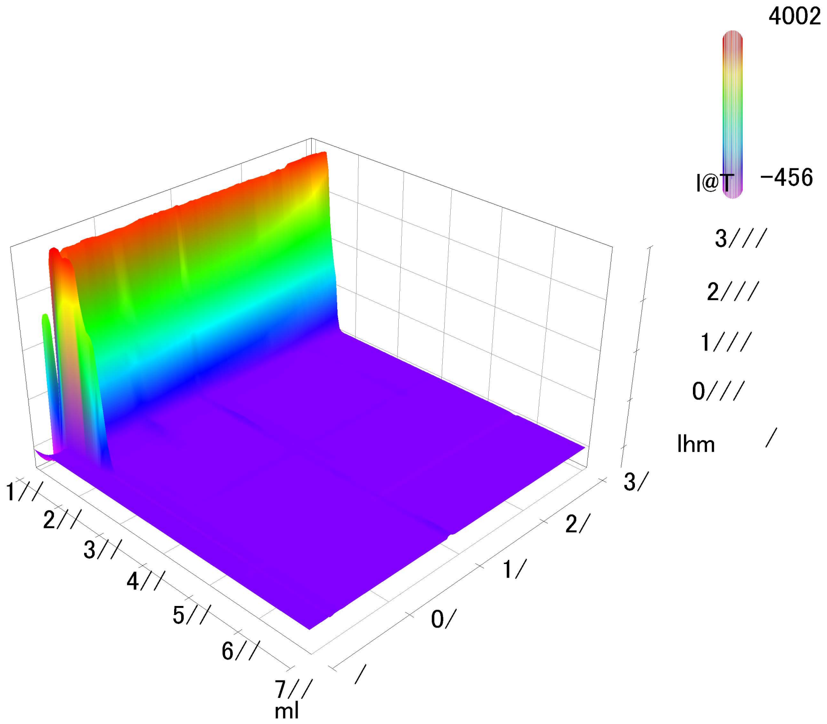

<Contour>

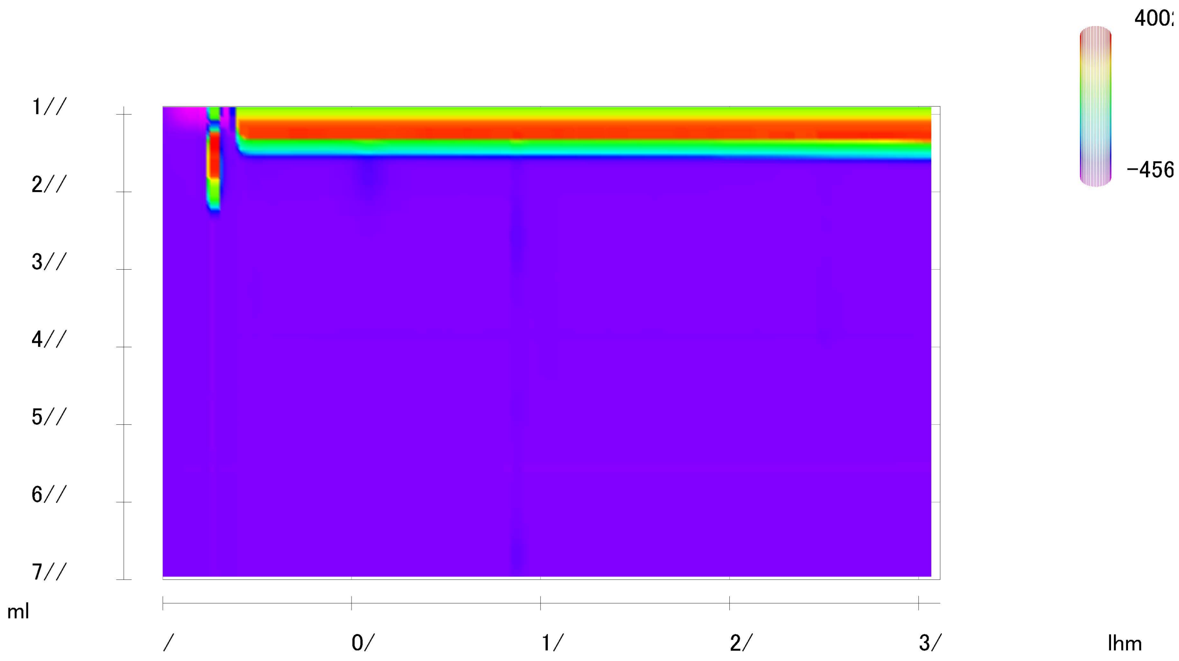

==== Shimadzu LCsolution Analysis Report ====

Sample Name : 2021-07-27 LH2 OP
Sample ID : 2021-07-27 LH2 OP
Operator : Admin
Data File Name : F:\Sascha ВЭЖХ\2021 ВЭЖХ\2021-07-27 LH2 OP.lcd
Method File Name : F:\Method\190-800.lcm
Batch File Name :
Report File Name : Default.lcr
Acquisition Date : 21/07/27 10:54:54
Modified Date : 22/05/22 23:49:11

<Chromatogram>

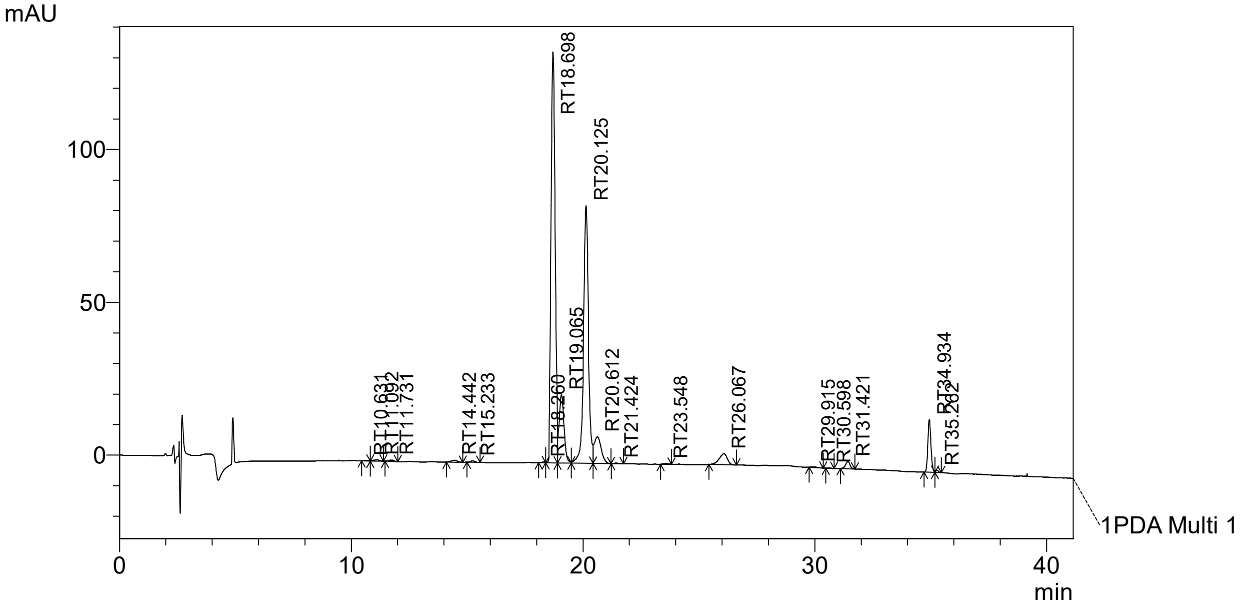

1 PDA Multi 1/360nm - 800nm 4nm

<Results>

PDA

ID#	Name	Ret. Time	Area	Minimum Peak Purity Inde	Conc.	Units
1	RT10.631	10.631	2472	Not calculated	0.000	mg/L
2	RT11.092	11.092	7170	Not calculated	0.000	mg/L
3	RT11.731	11.731	6722	Not calculated	0.000	mg/L
4	RT14.442	14.442	10699	Not calculated	0.000	mg/L
5	RT15.233	15.233	6513	Not calculated	0.000	mg/L
6	RT18.260	18.260	2436	Not calculated	0.000	mg/L
7	RT18.698	18.698	1675625	Not calculated	0.000	mg/L
8	RT19.065	19.065	333457	Not calculated	0.000	mg/L
9	RT20.125	20.125	1256682	Not calculated	0.000	mg/L
10	RT20.612	20.612	183818	Not calculated	0.000	mg/L
11	RT21.424	21.424	3152	Not calculated	0.000	mg/L
12	RT23.548	23.548	3012	Not calculated	0.000	mg/L
13	RT26.067	26.067	88493	Not calculated	0.000	mg/L
14	RT29.915	29.915	3447	Not calculated	0.000	mg/L
15	RT30.598	30.598	1344	Not calculated	0.000	mg/L
16	RT31.421	31.421	31786	Not calculated	0.000	mg/L
17	RT34.934	34.934	159021	Not calculated	0.000	mg/L
18	RT35.262	35.262	5286	Not calculated	0.000	mg/L
