# Supplementary material for: Simultaneous functioning of different light-harvesting complexes—a strategy of adaptation of purple bacterium Rhodopseudomonas palustris to low illumination conditions
Source: PeerJ. 2023 Jan 31;11:e14769. doi: 10.7717/peerj.14769 (PMC9897067; doi:10.7717/peerj.14769)
Supplement: Supplemental Information 6 [file peerj-11-14769-s006.pdf]

==== Shimadzu LCsolution Analysis Report ====

<3D Graph>

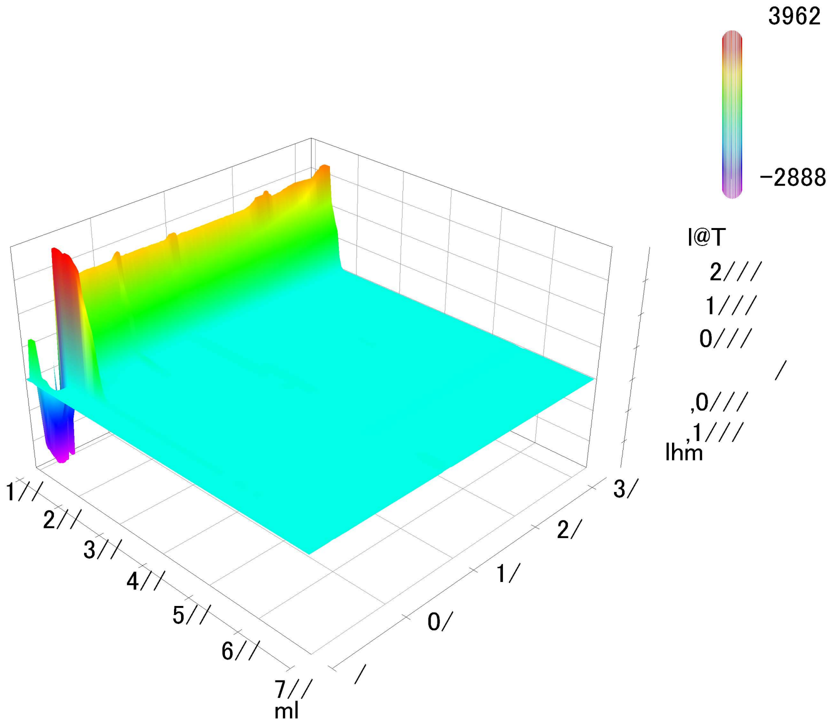

<Contour>

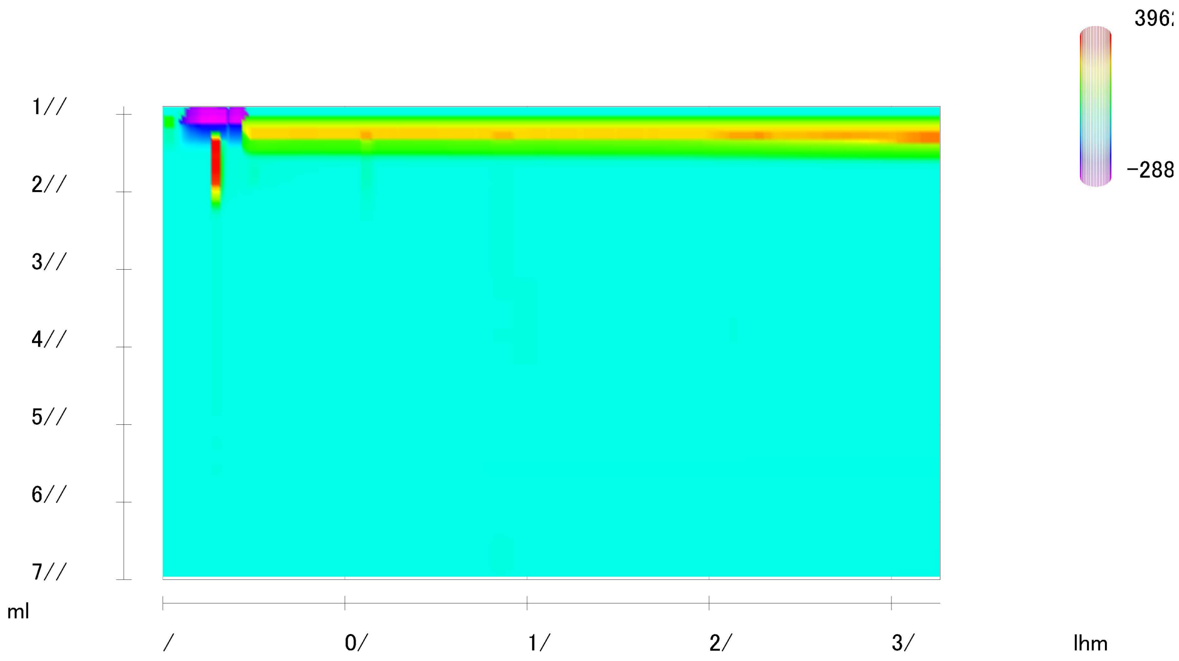

==== Shimadzu LCsolution Analysis Report ====

Sample Name	: 2021-07-27 LH4 OP
Sample ID	: 2021-07-27 LH4 OP
Operator	: Admin
Data File Name	: F:\Sascha ВЭЖХ\2021 ВЭЖХ\2021-07-27 LH4 OP.lcd
Method File Name	: F:\Method\190-800.lcm
Batch File Name	:
Report File Name	: Default.lcr
Acquisition Date	: 21/07/27 14:39:21
Modified Date	: 22/05/23 0:15:02

<Chromatogram>

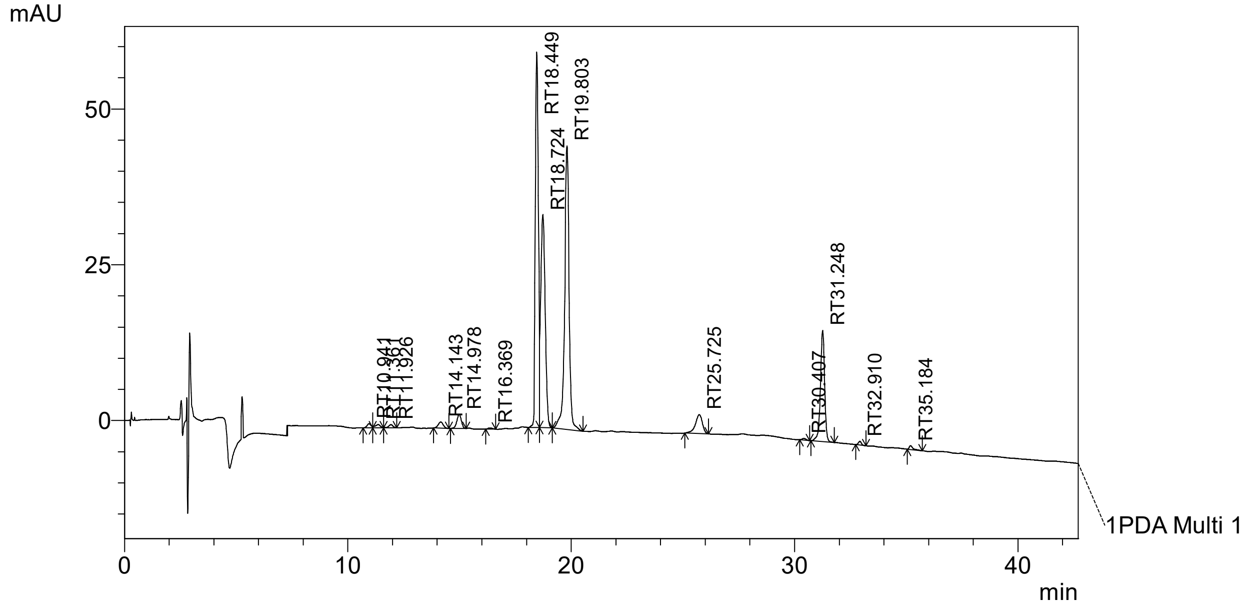

1 PDA Multi 1/360nm - 800nm 4nm

<Results>

PDA

ID#	Name	Ret. Time	Area	Minimum Peak Purity Inde	Conc.	Units
1	RT10.941	10.941	8597	Not calculated	0.000	mg/L
2	RT11.361	11.361	8747	Not calculated	0.000	mg/L
3	RT11.926	11.926	6688	Not calculated	0.000	mg/L
4	RT14.143	14.143	15874	Not calculated	0.000	mg/L
5	RT14.978	14.978	31440	Not calculated	0.000	mg/L
6	RT16.369	16.369	2372	Not calculated	0.000	mg/L
7	RT18.449	18.449	588722	Not calculated	0.000	mg/L
8	RT18.724	18.724	470230	Not calculated	0.000	mg/L
9	RT19.803	19.803	629297	Not calculated	0.000	mg/L
10	RT25.725	25.725	66035	Not calculated	0.000	mg/L
11	RT30.407	30.407	3181	Not calculated	0.000	mg/L
12	RT31.248	31.248	233829	Not calculated	0.000	mg/L
13	RT32.910	32.911	5610	Not calculated	0.000	mg/L
14	RT35.184	35.184	6925	Not calculated	0.000	mg/L
