# Supplementary material for: Simultaneous functioning of different light-harvesting complexes—a strategy of adaptation of purple bacterium Rhodopseudomonas palustris to low illumination conditions
Source: PeerJ. 2023 Jan 31;11:e14769. doi: 10.7717/peerj.14769 (PMC9897067; doi:10.7717/peerj.14769)
Supplement: File S1 [file peerj-11-14769-s007.pdf]

Supplementary File 1. Chromatograms of pigments from LL LH2 and LH4 complexes and chromatograms of standards used for pigment identification and quantification

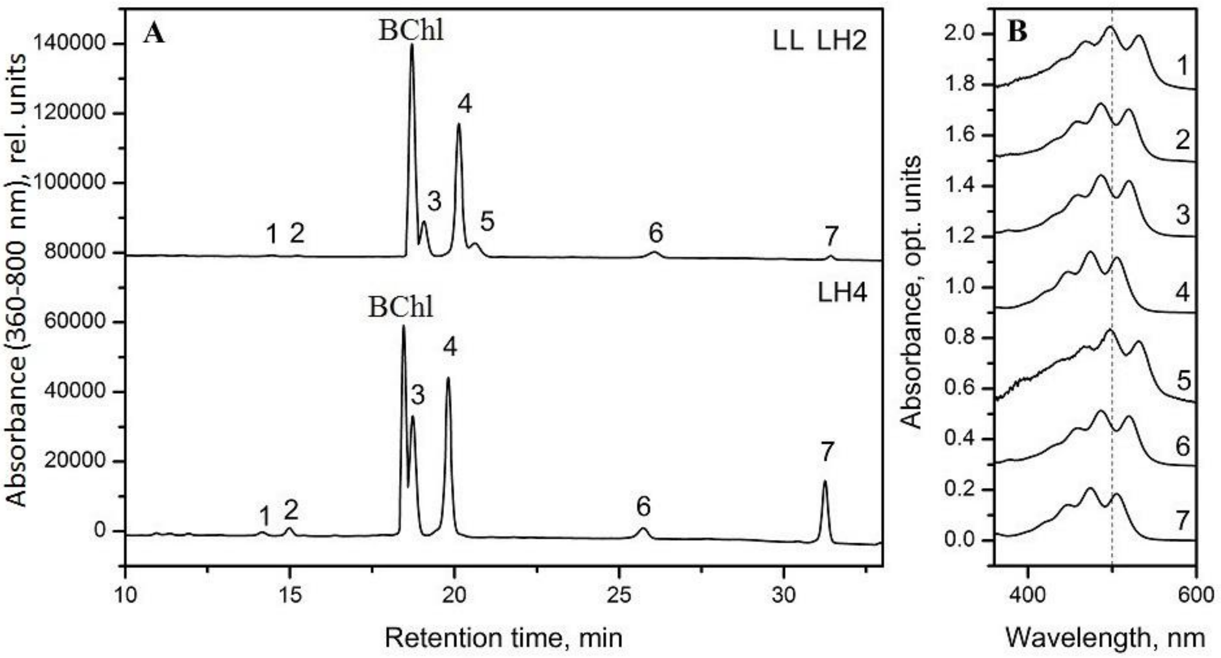

Fig. S1. A – the chromatograms of HPLC analysis of carotenoid compositon in LL LH2 and LH4 complexes from *Rps. palustris*. B – absorption spectra of individual carotenoid peaks indicated on the chromatogram. The HPLC analysis was carried out at a wavelength of 360-800 nm. The identification of the peaks: 1 – demethylspirilloxanthin, 2 – rhodovibrin, 3 – 3,4-didehydrorhodopin, 4 – rhodopin, 5 – spirilloxanthin, 6 – anhydrorhodovibrin, 7 – lycopene.

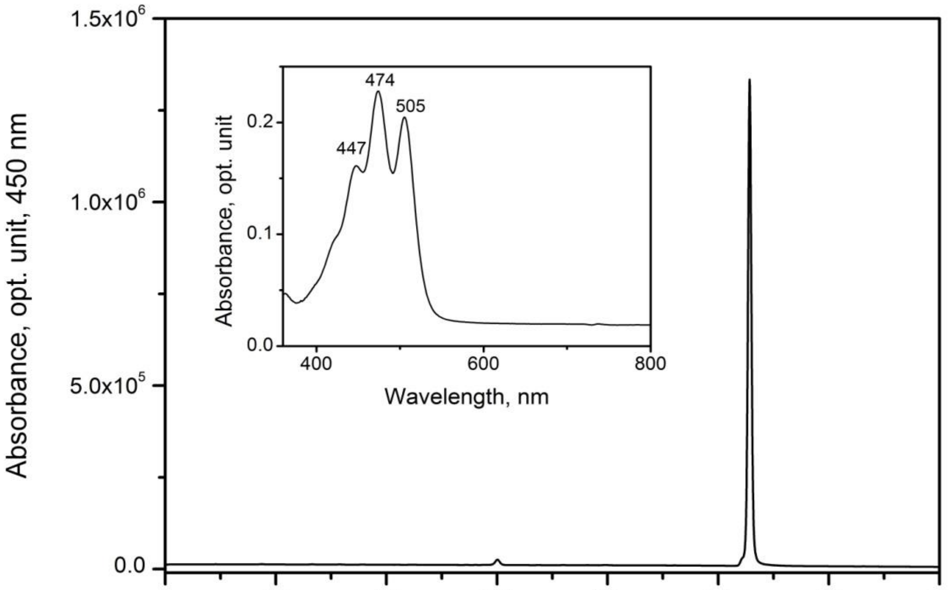

Fig. S2. Carotenoid standard of lycopene (Merck).

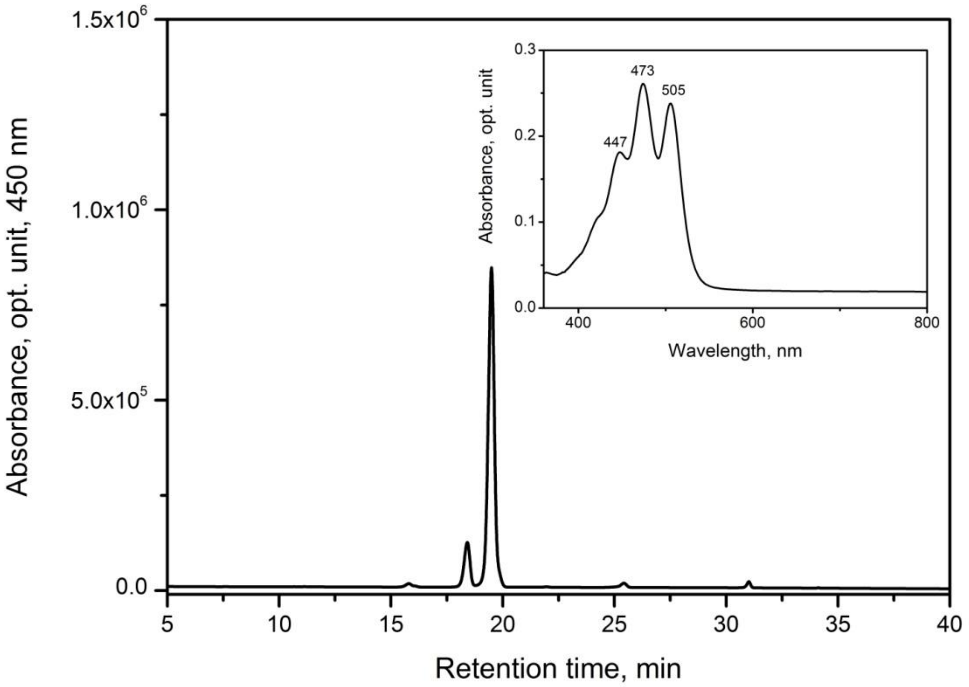

Fig. S3. Carotenoid standard of rhodopin from *Allochromatium vinosum*.

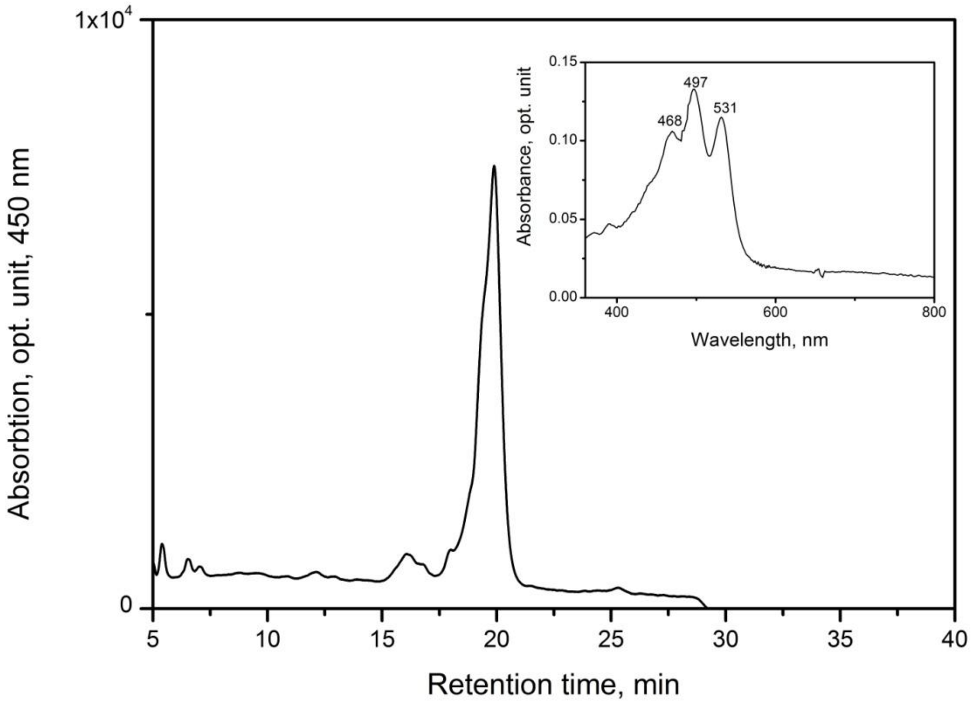

Fig. S4. Carotenoid standard of spirilloxanthin from *Rhodospirillum rubrum*.
